# Treatment with Epigallocatechin Gallate, Folic Acid, Vitamin B12, and Hyaluronic Acid Decreases HPV Positivity in Women Attending Regional Screening in Puglia

**DOI:** 10.3390/microorganisms12091897

**Published:** 2024-09-14

**Authors:** Andrea Tinelli, Sarah Gustapane, Martina Licchelli, Anna Chiara Coluccia, Gaetano Panese, Sara Proietti, Riccardo Gambioli

**Affiliations:** 1 Department of Obstetrics and Gynecology, CERICSAL (CEntro di RIcerca Clinico SALentino), Veris delli Ponti Hospital, 73020 Scorrano, Italy; 2 R&D Department, Lo.Li. Pharma, 00156 Roma, Italy

**Keywords:** HPV, cervical lesions, HPV DNA test

## Abstract

*Human Papillomavirus* (HPV) infection represents a global health concern. HPV infects the mucosa, particularly in the uterine cervix, where it may establish a persistent infection, exposing women to a risk of developing cancer. The available treatments include surgery or topic solutions, while a systemic treatment is still unavailable. In recent years, natural molecules such as epigallocatechin gallate (EGCG), folic acid (FA), vitamin B12, and hyaluronic acid (HA) gained importance as innovative therapies for HPV. We enrolled 163 women with a positive HPV DNA test or previous history of HPV infections, and a PAP test indicating LSIL/AGUS/ASCUS cytology. The patients in the treatment group (n = 86) received an oral combination of EGCG 200 mg, FA 400 µg, vitamin B12 1 mg, and HA 50 mg (1 cps/day) for 3 months (T1), while the control group (n = 77) underwent standard clinical surveillance. Both groups repeated a PAP test and an HPV DNA test after 3 (T1) months, and another PAP test after 6 months (T2) as a follow up. The treatment group experienced a significant reduction in HPV positivity at T1 compared to the control group. Moreover, the treatment group exhibited an improvement in cervical lesions either at T1 (*p* < 0.0001) or T2 (*p* < 0.00001).

## 1. Introduction

*Human Papillomavirus* (HPV) is a virus belonging to the *Papillomaviridae* family, which is a classification of double-stranded DNA viruses with a high tropism for human epithelia and mucosa.

Approximately 100 types of HPV can infect squamous epithelia, including the skin and mucosa of the upper respiratory and anogenital tracts [1], while approximately 40 genotypes can cause lesions of varying degrees in the cervical tissue and genital tract [1]. According to their oncogenic potential, all papillomaviruses are classified as low risk (LR), which usually lead to benign lesions (such as warts, condylomas, and recurrent respiratory papillomatosis) or precancerous lesions, and high risk (HR), which are highly associated with cancers of the lower genital tract, anus, and oropharynx both in men and women [2]. Therefore, HPV infection represents a burden to global healthcare, considering that it is the most common sexually transmitted infection (STI) worldwide [3].

After a histological diagnosis, the course of treatment for HPV-induced lesions is determined by their severity. Excisional methods, such as loop electrosurgical excision procedures (LEEPs), are typically used for moderate dysplasia and severe dysplasia/carcinoma in situ (HSIL/CIN2 or CIN3), whereas atypical squamous or glandular cells of undetermined significance (ASCUS/AGUS) and low-grade cervical dysplasia (LSIL/CIN1) do not require treatment at the time of diagnosis [4], and the standard medical approach is “wait and see” [5]. Nonetheless, depending on the local guidelines, LSIL may undergo preventive surgery in the absence of spontaneous healing or virus persistence for a long period of time. In Italy, for example, a history of two or more years of an LSIL is a risk factor for further malignant development of the lesion; thus, surgery is recommended in these cases. About 80% of people may have a natural reversion of the infection without any important clinical manifestation; however, 20% of patients may have a persistent infection, which could result in the development of cervical cancer (CC) [6]. Current data estimate that, in Italy, cervical cancer ranks as the fourth most frequent cancer among women aged between 15 and 44 years of age [7]. To identify and treat precancerous lesions before they develop into cancer, HPV vaccination and screening programs are the mainstays of cervical cancer prevention. 

According to European quality assurance guidelines, population-based screening programs that use evidence-based tests have the potential to significantly improve the target population’s health while also being the most appropriate and effective means of reducing the incidence and mortality of cervical cancer [8]. In Italy, the implementation of organized cervical screening programs within each region has been recommended since 1996 [9] and have been included in the Ministry of Health’s list of “Essential Health Interventions” since 2001 [10]. Given the role of certain types of HPV (high risk) in the development of cervical cancer, these regions have recently been invited to introduce HPV tests as first-level tests in cervical screening.

Despite these screening programs, no treatments for the virus itself or for its persistence exist [11]. Persistence is defined by some authors as infections with two consecutive positive HPV DNA tests [12]; others define persistence as the same viral strain remaining in the same patient for longer than nine months; and others define persistence as the amount of time it takes for removal. Despite all of these definitions, persistence represents a critical issue in HPV infection since it can lead to the growth of cancerous tumors [13]. In general, the capacity of the virus to evade the immune system is correlated with its persistence, and when HPV infection endures, genomic instability may enhance the integration of the virus’ genome into the host genome [14,15]. Once the virus has integrated, this leads to a breakpoint in the genetic sequence of the viral gene *E2*. *E2* is the HPV gene that, under physiological conditions, suppresses *E6* and *E7*, which are other HPV genes that are responsible for developing a proto-oncogenic environment. As such, the disruption of *E2* and the subsequent loss of function of the *E2* gene product result in the activation of the *E6* and *E7* viral oncogenes, which start exerting their oncogenic function at this point. This event promotes a downstream cascade of deregulated processes that ultimately leads to the accumulation of additional alterations in the host genome and result in the invasive cancer phenotype. For instance, E6 and E7 proteins impact the function of p53 and pRb proteins, two fundamental tumor suppressors [16,17]. Since HPV persistence is still an unsolved problem, research should concentrate on creating novel therapeutic approaches to address the infection. Natural molecules could be a good way to bridge this therapeutic gap. Recent research has shown that using folic acid (FA), hyaluronic acid (HA), vitamin B12 (B12), and epigallocatechin gallate (EGCG) can effectively treat HPV infections and prevent them from recurring [18]. Therefore, the aim of this study was to test the effect of an oral combination of EGCG, FA, B12, and HA in clearing HPV infection in women attending Puglia’s regional HPV screening program.

## 2. Materials and Methods

This study was an open-label controlled clinical trial. Participants were recruited during Puglia’s regional HPV screening program from November 2023 to January 2024. In the investigation, all patients aged from 20 to 45 years old were screened for the study.

The women whose results indicated positivity to HPV infection and who had at least a PAP test indicating LSIL/AGUS/ASCUS cytology were offered the chance to be enrolled in the investigation, which was conducted according to the ethical principles of the Declaration of Helsinki. The study was approved by the local IRB at the “Veris delli Ponti Hospital”, Scorrano, Lecce, Italy, and registered on ClinicalTrials.gov, Ref. No. NCT06098456.

All patients attending the cervicovaginal screening clinic were advised of the possibility of joining the study, and those who agreed to participate filled out the informed consent form. The patients’ data were recorded in a register and the patients’ name and surname were anonymized. Patients who agreed to participate in the study received an investigation participation number, which was communicated at the time of signing the consent form. All of the results of the screening tests were reported by the clinicians on a PC using the Excel program.

The exclusion criteria were (1) HPV-related pathologies; (2) ASC-H and HSIL (complications apart from LSIL or ASCUS); and (3) a diagnosis of cervical cancer. 

The enrolled patients were divided in two groups: a control group and a treatment group. 

The control group followed regional guidelines and repeated a PAP test and an HPV DNA test after 3 (T1) months, and another PAP test after 6 months (T2) as a follow up. The treatment group participants were offered the possibility of taking an oral combination of 200 mg EGCG, 400 µg FA, 1 mg vitamin B12, and 50 mg HA (1 cps/day) for 3 months (T1) (Pervistop^®^, Farmares s.r.l., Rome, Italy). Also, the treatment group repeated a PAP test and an HPV DNA test after 3 (T1) months, and another PAP test after 6 months (T2) as a follow up. 

The primary outcome of the study was to evaluate the clearance of HPV infection, determined by HPV DNA-negative test results. The secondary outcomes were the improvement or disappearance of lesions. The data were analyzed via a Chi-square test to evaluate the differences in the distribution between the two groups.

### 2.1. Cobas^®^ HPV Test

HPV positivity was assessed via a cobas^®^ HPV test (Roche^®^ Diagnostics, Rotkreuz, Switzerland). The cobas^®^ HPV test is a clinically validated and FDA-approved in vitro test that specifically identifies a pool of HR-HPV. This pool includes HPV16, HPV18, and other high-risk types (31, 33, 35, 39, 45, 51, 52, 56, 58, 59, 66, and 68) at clinically relevant infection levels. 

The cobas^®^ HPV test for use on cobas^®^ 5800/6800/8800 Systems delivers reliable, clinically validated assay performance for automated cervical cancer screening. Briefly, these systems utilize the amplification of target DNA via polymerase chain reaction (PCR) and nucleic acid hybridization for the detection of high-risk types in a single analysis. HPV DNA tests have extensive longitudinal data to support the safety of a negative result, and to ensure confidence in a negative result, each cobas^®^ HPV test also includes appropriate controls to verify that human cells are present in the sample.

All tests were performed by trained sanitary personnel at the “Veris delli Ponti Hospital”, Scorrano, Lecce, Italy, according to the manufacturer’s instructions.

### 2.2. Cervical–Vaginal Cytology (PAP Test)

The cervical–vaginal cytology examination is primarily a cervical cancer screening test. This test consists of a procedure that gently removes cells from the surface of the cervix and the surrounding area, and then microscopical analysis is performed. The PAP test was performed by the same gynecologist during the regional screening program at the “Veris delli Ponti Hospital”, Scorrano, Lecce, Italy. During the PAP test, the swab was transferred to a glass microscope slide, fixed in 95% ethanol, and treated using the Papanicolaou solution 2a orange G (Sigma-Aldritch, Saint Louis, MI, USA). The same anatomical pathologist examined all of the glass slides and classified the results according to the Bethesda System.

### 2.3. Statistical Analysis

The data were analyzed using GraphPad software (version 8.0.1, La Jolla, CA, USA). A Chi-square test was used for the analysis between pre and post treatments and the analysis between the treatment and the control group. A multivariate post hoc analysis was carried out to determine preventive and risk factors. A *p*-value of ≤0.05 was considered statistically significant. An independent statistician examined the results for accuracy.

## 3. Results

We enrolled a total of 163 women: 77 in the control group and 86 in the treatment group. Table 1 summarizes the patients’ data concerning age, BMI, number of partners, use of condoms, use of the pill, previous pregnancies, and previous abortions. Concerning these data, we did not find any significant differences in the baseline characteristics between the intervention and control group. Moreover, we found that the two groups were homogeneous regarding educational level (*p* = 0.940374), family status (*p* = 0.950030), concomitant diseases (*p* = 0.727134), and symptoms associated with the infection (*p* = 0.952419), guaranteeing an adequate starting sample without heterogeneity.

### 3.1. Positivity

**Figure 1:**
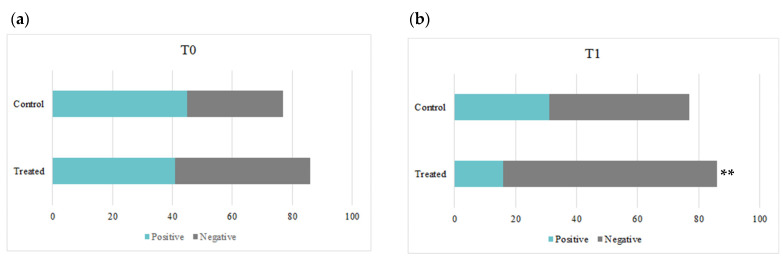
The graphs represent a comparison of HPV positivity between the control and treatment groups at baseline (T0) and after treatment (T1). (**a**) At T0, both the control and treatment groups showed similar levels of HPV positivity. (**b**) After 3 months of treatment (T1), the treatment group showed a significant decrease in HPV positivity compared to the control group (** *p* < 0.01).

At baseline (T0), we found that no statistical difference existed in terms of HPV positivity between the two groups. In the treatment group, 47.6% of women were positive at T0, while the remaining 52.3% were negative. In the control group, 58.4% of the patients were positive and the other 41.6% were negative at baseline. As depicted in Figure 1, the treatment group experienced a significant reduction in HPV positivity after three months (T1) when compared to the control group in the same period (*p* < 0.01). In fact, the percentage of women testing positive in the treatment group at T1 was 18.6%, while the percentage in the control group at the same timepoint was 40.3%. When comparing the intra-group variations between timepoints, the treatment group underwent a significant reduction in positivity (from 47.6% to 18.6%, *p* < 0.001), and the control group showed a similar trend (from 58.4% to 40.3%, *p* < 0.05).

A post hoc multivariate analysis determined the risk and preventive factors, as reported in Table 2. Age and BMI had no influence on the outcome of the treatment; condom use was found to be a preventive factor, as the treatment showed a higher efficiency in this population, while multiple partners, the usage of contraceptive pills, and previous pregnancy appeared to be risk factors for lower efficiency of the treatment in our study.

### 3.2. Cytology

**Figure 2:**
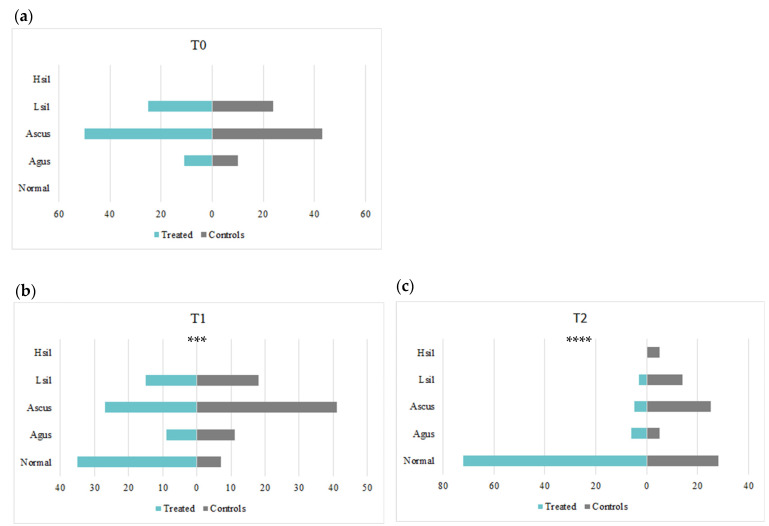
The graphs represent a comparison of cervical lesions in the control and treatment groups at baseline (T0), after 3 months (T1), and at 6 months (T2). At baseline (T0), both control and treatment groups showed similar cytology of cervical lesions (**a**). After 3 months of treatment (T1) and 6 months (T2) (**b**,**c**), the treatment group showed a significant improvement in cervical lesions (*** *p* < 0.0001 and **** *p* < 0.00001).

At baseline (T0), all patients enrolled in the study had cervical lesions with LSIL/AGUS/ASCUS cytology, while none had HSIL cytology. In the intervention group, 12.8% of patients had AGUS cytology, 58.1% of patients had ASCUS, and 29.1% of patients had LSIL. On the other hand, in the control group, 13.0% of patients had AGUS, 55.8% had ASCUS, and 31.2% had LSIL (*p* = 0.95 versus intervention group). Treatment with the oral combination of EGCG, FA, vitamin B12, and HA significantly improved the occurrence and manifestations of cervical lesions in a time-dependent manner; in fact, as presented in Figure 2, at T1, 40.7% of patients in the intervention group had normal cytology, 10.5% had AGUS, 31.4% had ASCUS, and 17.4% had LSIL. Conversely, 9.1% of patients in the control group achieved normal cytology, while 14.3% of patients exhibited AGUS cytology, 53.2% ASCUS, and 23.4% LSIL (*p* < 0.001 versus the intervention group). After 6 months (T2), 83.7% of patients in the control group had normal cytology, 7.0% had AGUS, 5.8% had ASCUS, and 3.5% had LSIL. In the control group 36.4% of patients had normal cytology, 6.5% had AGUS, 32.5% had ASCUS, 18.2% had LSIL, and 6.5% developed a HSIL cytology (*p* < 0.001 versus the intervention group).

## 4. Discussion

This clinical trial corroborated the effectiveness of the combined oral treatment of EGCG, FA, vitamin B12, and HA in decreasing positivity to HPV infection, and confirmed the positive effect of such a combination on the improvement of cervical lesions. Our data are in line with previous clinical evidence, in which HPV positivity decreased after four months [19] or after six months of treatment [20]. In a young patient with 9 years of HPV persistence, negativity to HPV was achieved after only 2 months of treatment. In that case, due to the severity of the situation, the patient took two caps/day [21]. The effectiveness of this treatment represents an important result, since, to date, no other medical tools have been effective in clearing HPV infection [22]. 

In our study, no one in either group had HSIL at baseline. After 3 (T1; *p* < 0.0001) and 6 months (T2; *p* < 0.00001), the oral association of EGCG, FA, vitamin B12, and HA improved cervical lesions, while in the control group, five women (6.5%) developed HSILs after six months. When examining the complete data distribution at six months, there were statistically significant differences (*p* < 0.00001) between the two groups. 

Patients with HPV-positive and low-grade lesions are typically sent to follow-up care in accordance with regional screening criteria. In general, about 80% of people with low-grade lesions may have a natural reversion of the infection without any important clinical manifestation; however, 20% of patients may have a persistent infection, which can lead to a worsening of lesions and may develop into cervical cancer over time. This method demonstrates the gap that currently exists in clinical practice and HPV infection care to combat its persistence [23]. Consequently, we examined an oral mixture of natural molecules for the first time as part of a regional screening program. 

The scientific community has previously concentrated on the characteristics of each molecule, showing how each one may play a significant role in HPV infection. 

EGCG interferes with the HPV life cycle by suppressing the oncogenes and oncoproteins E6/E7, which are responsible for viral oncogenic activity and cancer development [24]. The suppression of E6/E7 proteins correlates with the upregulation of tumor suppressor genes such as *p53*, *pRb*, and *p21* [25]. EGCG has an antiproliferative and proapoptotic action in cervical-cancer-derived cell lines [26,27,28]. Moreover, other studies have already demonstrated that EGCG can stimulate the interferon (IFN) pathway, which is one of the escape mechanisms of HPV, thus reinforcing the innate antiviral immunity against HPV [29].

Topical pharmaceutical preparations containing EGCG have already demonstrated positive efficacy for the treatment of external genital warts and perianal warts. Several studies [30,31] have, indeed, proven that EGCG induces a statistically significant clearance of all baseline EGW and anogenital warts when compared to placebo, leading to a great improvement and even the disappearance of the external lesions. Moreover, oral EGCG is potentially protective for patients with HPV-infected cervical lesions or cervical intraepithelial neoplasia, thus reducing the risk of cervical cancer [32]. 

Folic acid and cobalamin, known as vitamin B9 and vitamin B12, respectively, are water-soluble vitamins and crucial micronutrients, usually introduced with dietary sources, whose deficiency may correlate with different pathological conditions, including cancer [33]. They have a peculiar role in DNA synthesis and repair, and both are necessary for the synthesis of S-adenosylmethionine, which acts in various methylation reactions as the main donor of methyl groups. Low levels of folic acid decrease DNA methylation and, consequently, increase the frequency of fragile sites on DNA [34,35]. Moreover, folic acid and vitamin B12 inversely correlate with homocysteine levels, increased risk of cervical cancer, and vaginal HR-HPV infections [36,37]. Clinical studies demonstrated that, in the case of folate deficiency, persistent HPV infection and the progression of cervical dysplasia increase [38]. Moreover, subjects with higher folate and vitamin B12 status are 73% less likely to test positive for HR-HPV types and are more likely to test negative [38,39]. Therefore, both folate and vitamin B12 are necessary to maintain genomic stability, thus preventing the integration of HPV into cells’ genome. 

HPV infection is mainly acquired through sexual intercourse and the virus spreads through skin-to-skin contact, typically in areas of trauma and minor injuries. The loss of continuity in epithelia and mucosa allows the virus particles to penetrate and infect [40]; therefore, restoring the integrity of cervical tissue may represent a way to prevent HPV infections. HA, the main component of the extracellular matrix in epithelial and connective tissues, may represent a valid approach in this context. HA has several properties depending on its molecular weight [41,42]. In particular, very-low-molecular-weight hyaluronic acid (<5 kDa) and low-molecular-weight hyaluronic acid, by stimulating the production of pro-inflammatory factors, can promote the process of wound-healing repair [43,44]. Therefore, they have been extensively used in the gynecological field to increase vaginal epithelial thickness in postmenopausal women [45]. Moreover, a recent study revealed that supplementing HA in combination with other compounds may increase viral clearance and reduce the persistence of LSIL/CIN1 lesions [46].

A recently published in vitro study highlighted, for the first time, the synergism of EGCG, folic acid, vitamin B12, and HA in increasing apoptosis in HPV-positive cervical cancer cells (HeLa cells) by upregulating p53 and downregulating E6/E7 expression, respectively. In particular, treating this model of HPV persistence with these molecules had a stronger effect on apoptosis and p53 and E6/E7 expression levels than treatments with individual molecules [47].

In addition to these findings, recently, researchers have highlighted how the combined action of these molecules could also have a synergistic effect in counteracting HPV infection in clinical studies [19,20,21].

In particular, a previous case report has revealed, for the first time, the effectiveness of such a combination in improving cytological lesions in a young patient with 9-year HPV persistence, thus preventing or delaying hysterectomy surgery [21].

Furthermore, a pilot clinical study by Aragona and colleagues highlighted that 17 out of 20 women treated with combined EGCG, folic acid, vitamin B12, and HA achieved full viral clearance and showed no cytological or histological evidence of lesions following the treatment. In this way, the authors highlighted the applicability of these natural molecules in improving HPV clinical manifestations and persistence [19].

A recent case report analyzed five more clinical cases with positive HPV DNA tests and with cytological lesions of different grades. The authors reported that dietary supplementation with EGCG, FA, vitamin B12, and HA restored the negativity to viral DNA and improved clinical cytological conditions and related phlogosis after 6 months. In particular, one patient with HPV anal positivity became negative after 3 months of taking the dietary supplement at the dosage of 2 cps/day, thus also revealing, for the first time, the effectiveness of the oral combination of natural molecules in an anatomical region different from the cervical one [20].

Our study has some limitations: no randomization was performed, and genotyping or viral load quantification was not possible due to the type of instrumentation used for regional screening. 

Nevertheless, this was the first time in which women underwent this kind of treatment as part of a regional screening program. Moreover, our data confirmed previously published results in which the same oral combination significantly reduced the rate of HPV infection and improved cervical lesions [19]. 

## 5. Conclusions

Worldwide, the lack of medical intervention aimed at treating HPV infection continues to be a major medical concern. There are no effective therapeutic methods to eradicate HPV’s viral load, even with screening programs and vaccinations. Persistence is the most important risk factor for tumor development, as it exposes lesions and healthy tissues to a continuous oncogenic stimulus, and still represents a serious clinical challenge.

In a group of women enrolled in Puglia’s regional screening program, this clinical trial demonstrated that an oral combination of EGCG, FA, vitamin B12, and HA dramatically reduced HPV positivity and concurrently improved cervical lesions. We anticipate that additional research will confirm our findings, indicating that the care of women with HPV infection may benefit from the integration of this natural therapeutic strategy.

## Figures and Tables

**Table 1:** Baseline characteristics of patients enrolled in the study.

Parameters	Treatment Group	Control Group	*p*-Value
Age	25.4 ± 4.7	26.2 ± 4.6	0.319735
BMI	23.7 ± 2.3	23.9 ± 2.2	0.683126
Partners	1.36 ± 0.53	1.39 ± 0.54	0.729371
No condom [n (%)]	58 (67.4%)	54 (70.1%)	0.711748
No pill [n (%)]	58 (67.4%)	50 (65%)	0.368483
Previous pregnancies	0.71 ± 0.91	0.97 ± 1.09	0.095590
Previous abortion	0.33 ± 0.60	0.57 ± 0.79	0.183154

**Table 2:** Results of the multivariate post hoc analysis highlighting preventive and risk factors.

Parameters	Odds Ratio	95% CI
Condom use	0.486	[0.237–0.999]
Multiple partners	2.713	[1.098–6.702]
Use of contraceptive pills	8.606	[3.200–23.141]
Previous pregnancies	2.068	[1.086–3.937]

## Data Availability

The authors of the study are custodians of the data in anonymous form, which can possibly be provided to anyone who makes a motivated and reasoned request.
